# Workplace destruction and mental health among local nurses 13 months after the Great East Japan Earthquake: associations with PTSD and depression

**DOI:** 10.1539/eohp.2025-0033

**Published:** 2026-06-11

**Authors:** Yoko Takahashi, Masako Kakizaki, Atsushi Sakuma, Ikki Ueda, Mikika Abe, Ayami Nagao, Hatsumi Yoshii, Hidemitsu Saito, Kazunori Matsumoto

**Affiliations:** 1 Department of Nursing, Miyagi University, Miyagi, Japan; 2 Department of Health and Psychosocial Medicine, Aichi Medical University School of Medicine, Aichi, Japan; 3 Sendai Medical Center, Sendai, Japan; 4 Sendai Kakyoin Clinic, Sendai, Japan; 5 Department of Nursing, Sendai Seiyo Gakuin University, Sendai, Japan; 6 Faculty of Medicine University of Miyazaki Hospital, Miyazaki, Japan; 7 Division of Psychiatric Nursing Health Sciences, Tohoku University Graduate School of Medicine, Sendai, Japan; 8 Midorigaoka Hospital, Miyagi, Japan; 9 Mental Clinic OASIS, Sendai, Japan

**Keywords:** depression, disasters, nurses, posttraumatic stress disorder, workplace

## Abstract

**Objectives:**

Disaster mental health research has largely focused on personal losses, while workplace-related losses remain underexplored. The Great East Japan Earthquake of 2011 caused widespread devastation, including hospital destruction. This study examined the association between workplace destruction, posttraumatic stress disorder (PTSD), and depression among local nurses 13 months after the disaster.

**Methods:**

A cross-sectional survey was conducted with 414 nurses working in coastal Miyagi Prefecture at the time of the earthquake (response rate: 87.5%). PTSD and depression were assessed using the PTSD Checklist–Specific Version (PCL-S) and the Patient Health Questionnaire-9 (PHQ-9). Group differences were analyzed using the chi-square and t-tests. Logistic regression models estimated odds ratios (ORs) for workplace destruction after adjusting for age, work role, and disaster-related personal factors.

**Results:**

The prevalence of probable PTSD and depression were 11.7% and 24.6%, respectively. Nurses from tsunami-destroyed hospitals showed higher crude prevalence of severe symptoms. Workplace destruction demonstrated elevated odds in the baseline models; however, after full adjustment for personal and reconstruction-related burdens, the magnitude of the association was attenuated, and confidence intervals indicated statistical uncertainty. Family member death or missing status remained the strongest independent correlate of severe PTSD (adjusted OR 5.4; p<0.001).

**Conclusions:**

Workplace destruction was associated with adverse mental health outcomes in the crude analyses, but its independent contribution diminished after accounting for cumulative personal and disaster-related burdens. These findings suggest that long-term mental health vulnerability among disaster-exposed nurses reflects complex interactions between occupational disruption and personal loss rather than structural workplace damage alone.

## Introduction

The Great East Japan Earthquake (GEJE) struck northern Japan on March 11, 2011, with a magnitude of 9.0. The earthquake triggered a massive tsunami that claimed more than 18,000 lives and destroyed approximately 400,000 homes. In Miyagi Prefecture, located closest to the epicenter, over 10,000 people died, and coastal cities and towns were devastated. The disaster also affected medical facilities that are typically regarded as safe. Among the 2,832 medical institutions in Miyagi, 136 (4.8%) were completely destroyed, and 380 (13.4%) were partially damaged. Although most facilities in the prefecture reopened within 1 year, only 70–80% of hospitals in the northern coastal areas resumed operations, and 88 medical facilities, including five hospitals with beds, ceased operations entirely^1)^. Medical staff from devastated hospitals were reassigned to shelters, temporary clinics, and other facilities to continue providing care.

Large-scale disasters disrupt communities by destroying infrastructure, homes, and workplaces, and impose long-lasting psychological and social burdens. Although first-time respondents are often the focus of disaster-related mental-health research, local workers play a crucial role in recovery and reconstruction. These workers must cope with diverse stressors over extended periods while balancing professional and personal demands^2,3)^. However, relatively little attention has been paid to long-term mental health status. Although large-scale disasters can destroy thousands of workplaces, few studies have investigated the mental health of local workers who survive such losses and continue to work in affected communities.

Hospital nurses constitute a critical subgroup of these workers. Even under normal conditions, nurses are at a high risk of occupational stress, including traumatic exposure, vicarious trauma, secondary traumatic stress, and burnout^4,5,6)^. In disaster contexts, local nurses face stress as both survivors and providers of essential healthcare. Previous studies indicated that nurses temporarily deployed in disaster zones are vulnerable to mental health problems during the acute phase^7,8)^. However, apart from a few Japanese reports suggesting that mental health problems among local nurses may persist long-term^9)^, there is limited evidence of a prolonged impact on nurses who remain in disaster-affected areas.

This study examines the effect of workplace destruction on the prevalence of probable posttraumatic stress disorder (PTSD) and depression among local nurses approximately 13 months after the GEJE. We specifically investigate whether workplace destruction constitutes a distinct risk factor for severe PTSD and depressive symptoms, independent of disaster-related personal losses such as home destruction or the loss of family members. We hypothesize that workplace destruction would be associated with an increased risk of severe mental health problems in this population.

## Methods

### Study design

This cross-sectional study was conducted from March to May 2012 (almost 13 months after the earthquake) using a self-administered questionnaire distributed during mental health examinations conducted in each workplace.

### Participants

This research was conducted as part of a mental health support program for medical institutions in the affected areas by Tohoku University and the Miyagi Disaster Mental Health Care Center. The sample size was determined based on the number of nurses who participated in the support program. This study targeted hospitals that voluntarily requested workplace mental health screening and psychosocial support following the 2011 GEJE, reflecting screening service uptake intention rather than research-driven sampling; therefore, facilities were not randomly selected. Nurses were recruited from one public hospital in District A, one in District B, and three in District C, all of which are located in the adjacent coastal areas of Miyagi Prefecture, Japan. These areas were among the hardest hit by the earthquake and tsunami, where approximately 2% of the population was lost, and more than 30% of houses were destroyed, which is substantially higher than the prefecture-wide average (0.5% population loss, 9% total house collapse). Three of the five hospitals were destroyed by the tsunami, and their nurses were redistributed across mixed-duty stations, including other hospitals, evacuation centers, and temporary housing support sites at the time of the survey. A total of 473 nurses received questionnaires, of which 440 (90.9%) were returned.

Symptoms were screened using self-report instruments and logistic regression analyses. Model 3 employed entry-forced-entry adjustment, including all personal, disaster, relocation, and work-role reconstruction burdens for statistical independence inference. Nurses who began working after the disaster were excluded. Finally, 414 nurses who answered sufficient items to screen for probable PTSD and depression accurately were included (overall response rate: 87.5%).

Although nurses originally belonged to five hospitals, by the 13-month survey point, nurses from tsunami-destroyed hospitals had been widely reassigned across mixed-duty stations, whereas those from non-destroyed hospitals remained in their original facilities with minimal redistribution. Given the small number of facilities (n=5), we formally evaluated the magnitude of facility-level clustering before determining the analytical approach. To quantify clustering, we estimated the intra-class correlation coefficient (ICC) using a one-way analysis of variance, with facility as the grouping variable. The ICCs approached zero for both probable PTSD (ICC<0.01) and depression (ICC<0.01), indicating negligible between-facility variance at 13 months post-disaster. Given this minimal clustering effect and the small number of clusters, hierarchical random-intercept estimation using GLMM was not retained for primary inference to avoid unstable parameter estimation, while hospital clustering is acknowledged narratively to ensure design transparency.

### Assessments

The assessments included demographics (age and sex), work roles, disaster-related private life, and workplace risk factors. Private life risk factors include home destruction, displacement, and deceased/missing family member(s). Workplace risk was defined as hospital destruction or cessation of operations.

PTSD was measured using the Japanese version of the PTSD Checklist–Specific Version (PCL-S)^10)^. Probable PTSD was defined as ≥44; severe symptoms as ≥46. Depression was measured using the Japanese version of the Patient Health Questionnaire-9 (PHQ-9)^11)^. Probable depression was defined as ≥10; severe symptoms as ≥14. The cut-off values were based on thresholds validated in previous studies^12,13)^. The threshold of ≥46 has also been supported by a validation study after the Fukushima disaster, which reported that this cut-off showed good diagnostic accuracy for PTSD in Japanese populations^14)^. Importantly, a recent individual participant data meta-analysis demonstrated that a PHQ-9 threshold of ≥14, as well as the diagnostic algorithm, yielded prevalence estimates most consistent with the Structured Clinical Interview for DSM (SCID)^15)^. Internal reliability was confirmed in this study sample by calculating Cronbach’s α at the 95% confidence level using Advanced Statistics in SPSS Statistics 29 (IBM Corp, Armonk, NY, USA) (α=0.989 for PCL-S and α=0.979 for PHQ-9 in this sample).

Group differences were analyzed using the chi-square and t-tests. Logistic regression models estimated adjusted odds ratios (ORs) for workplace destruction, controlling for age (model 1) and age and work role (model 2). Model 3 was a fully adjusted, forced-entry logistic regression model that included age, role, disaster exposure, and private life factors. Model 4 additionally included a prespecified interaction term (workplace destruction × deceased or missing family member(s) for PTSD; workplace destruction × home destroyed for depression) to examine effect modification as an exploratory analysis. Since more than 90% of the participants were female, sex was not included as a covariate. Interaction effects between workplace and private life factors were explored, but were considered exploratory due to limited power. Missing data were handled using complete case analysis. Statistical analyses were performed with SPSS version 29.0. Statistical significance was set at *p*<0.05, which was considered significant.

### Ethical considerations

Data were acquired during mental health examinations conducted at each workplace. Electronic data were obtained without personal information. Therefore, written informed consent was not obtained from any of the participants. Instead, we disclosed the study information to the participants, including the objectives and procedures, and provided them with an opportunity to refuse to participate. The names of the administrative regions, cities, and towns were anonymized so that the participants’ workplaces were not specified. The participants’ rights and welfare were protected according to the ethical guidelines of the Declaration of Helsinki, and the ethical principles of the Ministry of Health, Labour and Welfare of Japan were upheld. The study protocol was reviewed and approved by the Ethics Committee of the Tohoku University Graduate School of Medicine.

## Results

Internal consistency of screening instruments in this study sample was excellent (PCL-S: Cronbach’s α=0.989, standardized α=0.991, 17 items; PHQ-9: Cronbach’s α=0.979, standardized α=0.981, 9 items), indicating high reliability.

Table 1 presents the demographic characteristics and the prevalence of mental health problems among the participants. High levels of both workplace- and private-life-related disaster damage were observed. Of the 414 nurses, 136 (32.9%) had their workplaces destroyed, 172 (41.8%) had their homes destroyed, and 45 (13.5%) had deceased or missing family member(s). The prevalence of home destruction was significantly higher in the workplace destruction group than in the non-destruction group.

**Table 1.** Basic characteristics of the participants

		Total (*N=*414)		Workplace destruction				*p*
				Destruction (*n=*136)		Non-destruction (*n=*278)		
		n	%	n	%	n	%	
Mean age (standard deviation)		42.2	(9.5)	42.4	(8.2)	42.1	(10.2)	0.728
Age group, years								
	20–29	42	10.4	6	4.5	36	13.4	0.001
	30–39	130	32.3	59	44.0	71	26.5	
	40–49	117	29.1	36	26.9	81	30.2	
	50–62	113	28.1	33	24.6	80	29.9	
Gender								
	Male	8	2.0	6	4.4	2	0.7	0.019
	Female	394	98.0	129	95.6	265	99.3	
Work role								
	Managerial positions	65	16.7	21	18.4	44	16.0	0.554
	Non-managerial positions	324	83.3	93	81.6	231	84.0	
Deceased or missing family member(s)								
	Yes	45	13.5	15	11.4	30	14.9	0.415
	No	289	86.5	117	88.6	172	85.1	
Home destroyed								
	Half collapsed or worse	172	41.8	75	56.0	97	35.0	<0.001
	Less than half collapsed	239	58.2	59	44.0	180	65.0	
Displacement								
	Yes	119	29.1	46	35.1	73	26.3	0.080
	No	290	70.9	85	64.9	205	73.7	
Workplace destruction								
	Destruction	136	32.9					
	Non-destruction	278	67.1					
PCL-S								
	Mean (standard deviation)	29.7	(11.0)	32.9	(10.8)	28.1	(10.8)	<0.001
	PTSD with cutoff score (≥44)	46	11.7	19	14.6	27	10.2	0.243
	Highest 10% of PCL-S (≥46)	39	9.9	18	13.8	21	8.0	0.074
PHQ-9								
	Mean (standard deviation)	6.8	(4.9)	7.4	(5.6)	6.5	(4.5)	0.130
	Depression with cutoff score (≥10)	100	24.6	37	27.2	63	23.3	0.396
	Highest 10% of PHQ-9 (≥14)	40	10.0	20	14.7	20	7.4	0.023

PCL-S, PTSD Checklist-Specific; PHQ-9, 9-item Patient Health Questionnaire; PTSD, post-traumatic stress disorder.

PCL-S: Data were missing for 20 participants

PHQ-9: Data were missing for 8 participants

The overall prevalence of probable PTSD (PCL-S ≥44) was 11.7%, with no significant difference between the destruction and non-destruction groups. The prevalence of severe PTSD symptoms was significantly higher in the destruction group (13.8%) than in the non-destruction group (8.0%). The mean PCL-S score was significantly higher in the destruction group, although the difference was modest.

The overall prevalence of probable depression (PHQ-9 ≥10) was 24.6%, with no significant group difference. The prevalence of severe depressive symptoms was significantly higher in the destruction group (14.7%) than in the non-destruction group (7.4%). The mean PHQ-9 scores did not differ significantly between the groups.

Table 2 shows the bivariate analyses of the participant characteristics and the prevalence of severe PTSD and depressive symptoms. Severe PTSD symptoms were significantly more common among those who had deceased or missing family member(s), and tended to be more common among those working at hospitals destroyed by the tsunami. Severe depressive symptoms were significantly more frequent among those working at destroyed hospitals, and tended to be more common among those whose homes were destroyed.

**Table 2.** Basic characteristics of the participants in relation to the prevalence of severe PTSD and depressive symptoms

		PCL-S <46		PCL-S ≥46		*p*	PHQ-9 <14		PHQ-9 ≥14		*p*
		*n*	%	*n*	%		*n*	%	*n*	%	
		355		39			366		40		
Mean age (standard deviation)		41.6	(9.6)	43.9	(8.0)	0.170	41.8	(9.6)	44.7	(8.1)	0.067
Age group											
	20–29	40	11.6	2	5.3	0.142	41	11.5	1	2.6	0.156
	30–39	117	33.9	10	26.8		119	33.5	10	25.6	
	40–49	95	27.5	17	44.7		98	27.6	15	38.5	
	50–60	93	27.0	9	23.7		97	27.3	12	33.3	
Gender											
	Male	4	1.2	4	8.1	0.022	5	1.4	3	7.7	0.035
	Female	341	98.8	34	91.9		351	98.6	36	92.3	
Work role											
	Managerial positions	57	17.0	5	13.9	0.815	55	16.1	8	20.5	0.495
	Non-managerial positions	279	83.0	31	86.1		287	83.9	31	79.5	
Deceased or missing family member(s)											
	Yes	33	11.9	11	28.9	0.010	37	12.7	6	17.1	0.432
	No	244	88.1	27	71.1		255	87.3	29	82.9	
Home destroyed											
	Half collapsed or worse	147	41.8	18	46.2	0.612	149	41.0	22	55.0	0.095
	Less than half collapsed	205	58.2	21	53.8		214	59.0	18	45.0	
Displacement											
	Yes	100	71.4	25	64.1	0.356	106	29.4	12	30.0	1.000
	No	250	28.6	14	35.9		255	70.6	28	70.0	
Workplace destruction											
	Destruction	112	31.5	18	46.2	0.074	116	31.7	20	50.0	0.023
	Non-destruction	243	68.5	21	53.8		250	68.3	20	50.0	

PCL-S, PTSD Checklist, Specific; PHQ-9, 9-item Patient Health Questionnaire; PTSD, post-traumatic stress disorder.

PCL-S: Data were missing for 20 participants

PHQ-9: Data were missing for 8 participants

chi–square tests or *t*–tests were used

Table 3 shows the adjusted ORs and 95% confidence intervals (CIs) of the logistic regression models. In crude comparisons, nurses exposed to workplace destruction had a higher proportion of high-risk PTSD and severe depression screening positives.

**Table 3.** Adjusted odds ratios and 95% confidence intervals for severe PTSD and depressive symptoms by workplace destruction, work factor, and disaster-related private-life risk factors

		Model 1					Model 2					Model 3				
		OR	95% CI			*p*	OR	95% CI			*p*	OR	95% CI			*p*
**Factors associated with severe PTSD symptoms (PCL-S ≥46)**																
Disaster-related workplace risk factors																
Workplace destruction	Destruction	1.89	0.96	to	3.73	0.065	1.88	0.92	to	3.85	0.083	1.23	0.60	to	2.52	0.569
Work factor																
Work role	Managerial positions	0.67	0.24	to	1.86	0.439	0.64	0.23	to	1.79	0.396	0.77	0.30	to	1.98	0.584
Disaster-related private-life risk factors																
Deceased or missing family member(s)	Yes	2.97	1.34	to	6.60	0.007	3.63	1.60	to	8.26	0.002	4.32	1.95	to	9.60	0.001
Home destroyed	Half collapse or worse	1.32	0.67	to	2.59	0.420	1.32	0.65	to	2.67	0.446	0.78	0.34	to	1.82	0.567
Displacement	Yes	1.58	0.78	to	3.20	0.204	1.61	0.77	to	3.36	0.202	0.74	0.44	to	1.25	0.261
**Factors associated with severe depressive symptoms (PHQ-9 ≥14)**																
Disaster-related workplace risk factors																
Workplace destruction	Destruction	1.99	1.02	to	3.89	0.044	2.31	1.16	to	4.58	0.017	1.50	0.87	to	2.58	0.144
Work factor																
Work role	Managerial positions	1.07	0.45	to	2.55	0.881	1.03	0.43	to	2.47	0.950	1.00	0.50	to	2.03	0.985
Disaster-related private-life risk factors																
Deceased or missing family member(s)	Yes	1.36	0.53	to	3.51	0.525	1.42	0.54	to	3.71	0.479	0.99	0.46	to	2.16	0.987
Home destroyed	Half collapse or worse	1.77	0.91	to	3.46	0.093	2.02	1.02	to	4.00	0.044	1.63	0.90	to	3.00	0.112
Displacement	Yes	1.15	0.56	to	2.37	0.708	1.24	0.60	to	2.58	0.566	1.26	0.82	to	1.94	0.290

CI, confidence interval; OR, odds ratio; PCL-S, PTSD Checklist, Specific; PHQ-9, 9-item Patient Health Questionnaire; PTSD, post-traumatic stress disorder.

Model 1: Adjusted for age

Model 2: Adjusted for age and work role

Model 3: Multivariate: Adjusted for age, work role, and disaster-related private-life risk factors. For disaster-related private-life risk factors

However, in the fully adjusted forced-entry logistic regression (model 3), workplace destruction was not an independent predictor of long-term PTSD (adjusted OR 1.23; 95% CI, 0.60–2.52; *p*>0.05) or severe depression (adjusted OR 1.50; 95% CI, 0.87–2.58; *p*>0.05).

Model 4 included a prespecified interaction term added only after full adjustment as an exploratory sensitivity analysis (PTSD: workplace destruction × deceased or missing family member[s]; depression: workplace destruction × home destroyed). No statistically significant effect modification was detected (*p*>0.05; 95% CI crossed 1 for the interaction), indicating the absence of a meaningful long-term interaction impact on the inference axes.

No statistically significant interaction effects were observed between workplace destruction and the private life risk factors for PTSD or depression. However, these analyses were considered exploratory and may have been limited by their statistical power.

## Discussion

This study examined the mental health of local nurses who experienced the GEJE and continued to be involved in long-term community recovery and reconstruction. Nurses from tsunami-destroyed hospitals showed a higher crude prevalence of severe mental health symptoms; however, these associations were attenuated after adjusting for personal and disaster-related factors. To our knowledge, this is the first study to investigate the prevalence of probable PTSD and depression among local nurses in relation to workplace destruction following a large-scale disaster.

The prevalence of probable PTSD in our sample (11.7%) was considerably higher than the 12-month prevalence of PTSD in the general Japanese population (0.7%)^16)^, suggesting an increased risk in this population. Our prevalence estimate was comparable to that of first responders in disaster settings (10–20%)^17,18)^. Furthermore, more than one in eight nurses in our study lost family members, and this loss was strongly associated with severe PTSD symptoms, consistent with prior research^19,20)^. These findings emphasize the need for interventions, including workplace-based programs, to address bereavement and grief in disaster-exposed populations.

Similarly, the prevalence of probable depression (24.6%) in our study was markedly higher than the 12-month prevalence of depression in the general Japanese population (3%)^16)^, higher than estimates from general population surveys (6.1–16.0%)^21,22)^, and from first respondents following disasters (10.8–21.7%)^8,23)^. Furthermore, our study was substantially higher than the general resident cohorts screened around 1 year post-GEJE^24)^, which showed that 9% scored ≥13 on the Kessler Psychological Distress Scale (K6). indicating the risk of severe mental illness among residents of the tsunami-affected Miyagi Prefecture. These results suggest that local workers, including nurses, who remain in disaster-affected areas are at a high risk for depression.

To examine whether workplace destruction independently contributed to severe PTSD symptoms at 13 months post-disaster, we conducted forced-entry logistic regression analyses. In age-adjusted and age–work-role–adjusted models, workplace destruction showed elevated odds of severe PTSD. However, in the fully adjusted model — including age, work role, household damage, relocation, and family bereavement — the association attenuated (adjusted OR 1.23; 95% CI, 0.60–2.52).

Although the point estimate remained above unity, the wide confidence interval indicated considerable statistical uncertainty, and the magnitude of the association was substantially smaller than that observed for family member loss. In contrast, deceased or missing family member(s) remained a strong independent correlate of severe PTSD (adjusted OR 5.4), consistent with previous disaster mental health literature emphasizing proximal traumatic loss as a primary driver of persistent PTSD symptoms.

A similar analytical approach was applied to severe depressive symptoms. In the baseline models adjusted for age and work role, workplace destruction was associated with elevated odds of severe depression. However, after full adjustment for personal demographics, household damage, relocation, and reconstruction-related burdens, the magnitude of the association decreased, and the confidence intervals widened. The adjusted odds ratio remained above 1 but with substantial imprecision, indicating statistical uncertainty rather than the definitive absence of an association.

This pattern suggests that workplace destruction may operate in conjunction with cumulative personal and reconstruction-related stressors rather than as an isolated determinant of chronic depressive symptoms. Depression in disaster-exposed populations is influenced by ongoing secondary stressors and life-reconstruction burdens, which may partially explain the attenuation observed in fully adjusted models.

Importantly, our study identified that chronic depression vulnerability in nurses who remained locally active during prolonged recovery was shaped primarily by cumulative personal, family, and reconstruction axis burdens, and not isolated structural damage at the original facility. The attenuation observed after adjustment suggests that workplace destruction was intertwined with broader personal and reconstruction-related burdens, rather than acting as an isolated determinant.

This insight contributes scarce nurse-specific evidence to the GEJE responding-worker mental health literature, demonstrating that chronic depressive risk should be supported psychosocially through age- and life-reconstruction occupational burden reduction policies, rather than through an isolated structural workplace recovery focus alone.

For depression, the interaction between workplace destruction and home destruction was not statistically significant (OR 0.67; 95% CI, 0.22–2.02). Similarly, for PTSD, the interaction between workplace destruction and family-member loss showed wide confidence intervals (OR 0.51; 95% CI, 0.10–2.68). These interval estimates indicate substantial uncertainty rather than a definitive absence of modification.

One plausible explanation for the persistence of probable depression among disaster-exposed nurses is that secondary stressors following structural workplace loss, rather than the destruction of the original facility alone, influence chronic mental health outcomes^25,26)^. Consistent evidence has shown that mental health problems among local GEJE responders, including healthcare workers, remained markedly elevated beyond the national baseline prevalence^27)^. Furthermore, the onset and persistence of depression were influenced not only by direct exposure to traumatic events but also by subsequent secondary stressors^28)^; depression is also known to persist longer after disasters than PTSD^29,30)^. In our sample of destroyed hospitals, workplace destruction initially appeared as an occupational risk signal, but fully adjusted enter logistic regression revealed OR elevation attenuation due to confounding household traumatic loss, transfers, relocation, work-role disruption, and cumulative reconstruction burdens^26,27)^. These findings indicate that structural workplace damage alone may not fully explain long-term depressive vulnerability when cumulative personal and recovery-related stressors are considered. This pattern provides additional nurse-specific insights for occupational disaster mental health planning, indicating that integrated strategies addressing grief support and occupational life burdens, particularly role disruption, transfer stress, relocation, and aging-associated cumulative vulnerability, are essential for protecting a stable nursing workforce during prolonged recovery^27)^.

### Strengths and limitations

Strengths: this study included rare and occupationally important nurses working in the destroyed hospitals. A high response rate (87.5%) enhanced representativeness and minimized the selection threat to the baseline inference. Workplace structural destruction was objectively predefined and retained as a key exposure signature, enabling an inferential examination of occupational disaster burden. Our analysis uncovered the confounding structure of workplace destruction for chronic depressive positives, rather than rejecting our hypothesis, thus providing new clarity on occupational risk patterns. The assessment of both workplace and private life reconstruction burdens and screening for PTSD and depression (not only PTSD, as in many prior studies) further extends the scope of existing GEJE responder mental health research.

Limitations: the sample size was modest; although the overall 414 samples satisfied minimum events-per-variable stability to enter main-effects inference (events per variableadequate for two severe screening outcomes), statistical power for interaction axes remained limited. The cross-sectional design of this study precludes causal inferences. Symptoms were self-reported rather than clinically diagnosed, possibly resulting in an overestimation of prevalence. Cut-offs, while validated, may imperfectly capture severity. The sample was 90% female, which limited the gender analysis. A complete case analysis may have introduced bias. Facility-based intra-class correlation (five hospital clusters coded a priori) was considered, but hierarchical random-intercept estimation (GLMM) was not retained due to sparse clusters and negligible inter-facility variance at a 1-year recovery phase; this clustering context is therefore acknowledged narratively for transparency without hierarchical inference. The findings reflect a 13-month post-disaster psychological recovery phase, and may not be generalizable beyond this specific chronic reconstruction period.

These strengths and limitations collectively demonstrate practice-relevant value for occupational disaster mental health strategies among redistributed nursing cohorts at 1 year, emphasizing attenuation after adjustment, grief intensity resolution, and aging-associated cumulative work–life reconstruction vulnerability support as core axes for workforce protection and sustainable disaster recovery.

In conclusion, although workplace destruction showed elevated crude associations with both PTSD and depression, its independent contribution was attenuated after adjusting for personal and disaster-related burdens, and the confidence intervals indicated statistical uncertainty. These findings suggest that long-term mental health vulnerability among disaster-exposed nurses is shaped by cumulative personal, household, and reconstruction-related stressors rather than by structural workplace loss alone. Interval estimates further indicate that modest associations cannot be excluded and should be examined in larger longitudinal studies.
